# Monocytes and Macrophage-derived mediators influence the behavior of squamous cell carcinoma cell lines

**DOI:** 10.1590/1678-7757-2025-0380

**Published:** 2025-11-17

**Authors:** Graziela PERRI, Raíssa Gabrieli CANDIDO, Luiz Henrique Camargo SOARES, Rafael Carneiro ORTIZ, Izabel de CAMARGO, Maria Renata Sales NOGUEIRA, Edgard José Franco MELLO, Ana Lucia COELHO, Edwin M POSADAS, Cory HOGABOAM, Karen A CAVASSANI, Ana Paula CAMPANELLI

**Affiliations:** 1 Universidade de São Paulo Faculdade de Odontologia de Bauru Departamento de Ciências Biológicas Bauru SP Brasil Universidade de São Paulo, Faculdade de Odontologia de Bauru, Departamento de Ciências Biológicas, Bauru, SP, Brasil.; 2 Secretaria de Estado da Saúde Instituto Lauro de Souza Lima Bauru SP Brasil Secretaria de Estado da Saúde, Instituto Lauro de Souza Lima, Bauru, SP, Brasil.; 3 Cedars-Sinai Medical Center Division of Pulmonary and Critical Care Medicine Department of Medicine Los Angeles CA USA Cedars-Sinai Medical Center, Division of Pulmonary and Critical Care Medicine, Department of Medicine, Los Angeles, CA, USA.; 4 Cedars-Sinai Medical Center Samuel Oschin Comprehensive Cancer Institute Los Angeles CA USA Cedars-Sinai Medical Center, Samuel Oschin Comprehensive Cancer Institute, Los Angeles, CA, USA.

**Keywords:** Macrophage, Monocytes, Squamous cell carcinoma

## Abstract

Immune cells play diverse roles in cancer development. Myeloid cells are key drivers of tumor-escape mechanisms as they suppress immune responses, facilitate metastasis, and contribute to therapy resistance. In particular, macrophages can be polarized into an inflammatory M1 (anti-tumor) or anti-inflammatory M2 (pro-tumor) phenotype. M2 macrophages are associated with tumor progression, as they secrete factors that promote tumor angiogenesis, suppress T-cell activity, and correlate with poor clinical outcomes in squamous cell carcinoma (SCC). Given this context, this study aims to demonstrate the biological effects of monocytes and both M1 and M2 macrophages in squamous cell carcinoma. Our data indicate higher CD163 immunoreactivity in biopsies from SCC patients. Furthermore, we found that a conditioned medium (CM) containing bioactive compound generated by M2 macrophages enhances the proliferation and invasion of the SCC-25 cell line in vitro. Surprisingly, CM derived from blood CD14^+^ monocytes increased SCC-25 proliferation at the same rate of M2 macrophages-CM. M1 macrophages conditioned medium significantly enhanced the motility and decreased proliferation in Detroit 562 cells. The analysis of tumor-associated transcripts showed that both M1 and M2 conditioned medium induced high levels of *EPCAM* mRNA and significantly decreased the expression of *MYC*, an epithelial-to-mesenchymal transition marker, in SCC cell lines. Detroit cells exposed to conditioned medium from monocytes and macrophage also showed elevated *SOX2* mRNA levels. The findings suggest that monocytes and macrophage mediators exert distinct biological effects on SCC cell lines.

## Introduction

Head and neck squamous cell carcinomas (HNSCC) are a heterogeneous group of malignant tumors that develop in the epithelial linings of the oral cavity, pharynx, and larynx. They exhibit distinctive histological and molecular features^1^ and are the most common cancers of the head and neck region.^2^ Epidemiological studies have identified a diverse range of risk factors for HNSCC, including alcohol and tobacco use, radiation exposure, underlying genetic disorders, exposure to environmental pollutants, and infections with human papillomavirus (HPV) or Epstein–Barr virus (EBV).^3^

Published studies have highlighted the essential role of the tumor microenvironment (TME) in providing a supportive niche that promotes SCC development and metastasis.^4^ Within the tumor microenvironment, there is considerable diversity in inflammatory components.^5^ However, tumor-infiltrating monocytes, macrophages, and dendritic cells are consistently found in the composition of the tumor microenvironment.^6,7^ Macrophages, among the most plastic leukocytes in the tumor microenvironment can be polarized into an inflammatory M1 (anti-tumor) or anti-inflammatory M2 (pro-tumor) phenotype.^8^ M1 macrophages are characterized by high expression of IL-6, IL-12, CXCL10, TNF, and inducible nitric oxide synthase (iNOS), whereas M2 macrophages are defined by surface expression of CD206, CD163, and CD36, along with high levels of TGF-β and IL-10.^9,10^ Polarizing tumor-associated macrophages (TAMs) toward the M1 phenotype has been shown to promote tumor regression in several cancer types, primarily by reversing the immunosuppressive niche within the tumor microenvironment.^11^ Conversely, several cancers contain M2-like macrophages that display suppressive activity,^6^ including increased angiogenesis, production of matrix metalloproteinase (MMPs), immune suppression, and tumor drug resistance.^12-15^

In the tumor microenvironment, diverse intrinsic and extrinsic mechanisms influence the infiltration and activity of tumor associated macrophages.^16^ As in most solid tumors, the macrophage balance in SCC tends to shift toward the M2 phenotype.^16^ Studies have shown that in HNSCC, TAMs are predominantly polarized to the M2 phenotype.^17^ Additionally, the density of M2 macrophages was positively correlated with the pathological stage of oral squamous cell carcinoma (OSCC).^18^ In contrast, HNSCC patients with high expression levels of the M2 marker CD163 have significantly worse clinical outcomes.^19^ We confirmed these findings by detecting CD163^+^ cells in human squamous cell carcinoma lesions. We aimed to evaluate the role of human CD14^+^ monocytes and macrophages in promoting and modulating the activity of SCC cells *in vitro*. Our findings indicated that M1-CM increased the motility of SCC-25 cells, while M2-CM and monocyte-CM significantly enhanced their proliferation. Conversely, M1-CM significantly increased motility and reduced proliferation in Detroit 562 cells, whereas monocyte- and M2-CM-treated cells showed no differences in proliferation compared with medium. Furthermore, monocyte, M1 and M2 conditioned media promoted a significantly increased the invasiveness of SCC-25 cells. The addition of both M1- and M2-CM upregulated *EPCAM* expression, an epithelial-to-mesenchymal transition marker, in SCC-25 and Detroit 562 cells, while downregulating *MYC* expression, a stemness marker, in both cell lines.

## Methodology

### SCC samples and healthy volunteers

To analyze the presence of macrophages in SCC samples, we reviewed patients with carcinomas that were surgically resected and originally diagnosed as SCC at Instituto Lauro de Souza Lima between 2014 and 2016. Tumor slides and blocks were available for histological and immunohistochemical evaluation from 14 patients. This retrospective study was approved by the Institutional Review Board of Instituto Lauro de Souza Lima (37644714.7.0000.5475). Blood specimens were collected from healthy volunteers after written informed consent, under Cedars Sinai Medical Center IRB-approved protocol (IRB #Pro00045523).

### Histopathology

Hematoxylin- and eosin-stained sections were reviewed by two pathologists to confirm the histopathological diagnosis. From each tumor specimen, formalin-fixed and paraffin-embedded samples were collected. All sections were analyzed under an optical microscope, and microphotographs were collected using a digital camera (Leica DFC310 FX, Leica Microsystems GmbH, Wetzlar, Germany).

### Immunohistochemistry

Immunohistochemical staining was performed on formalin-fixed, paraffin-embedded tissue sections. A human anti-CD68 monoclonal antibody (clone IR60961-2; Agilent, Santa Clara, CA, USA) and an anti-CD163 monoclonal antibody (clone M5E2, R&D Systems, Minneapolis, MN, USA) were used to analyze macrophage populations. Slides were deparaffinized, rehydrated, and rinsed in distilled water for 5 min. Endogenous peroxidase activity was blocked by incubation in 0.5% hydrogen peroxide in methanol. After washing, the slides were incubated with the primary antibody overnight at 4°C, washed with phosphate-buffered saline (PBS), and then incubated with the appropriate biotinylated antibody for 1 hours at room temperature. The staining was visualised using Impact DAB solution (Vector Laboratories), and the slides were counterstained with hematoxylin. Immunostained slides were scanned using the Aperio automated whole-slide scanning system (Aperio Scanscope CS Slide Scanner, Aperio Technologies Inc, Vista, CA, USA) and viewed using ImageScope software (Aperio Technologies Inc). CD68 and CD163 immunoreactivity were analyzed semi-quantitatively as the proportion of labelled cells. The staining intensity of each marker, analyzed by the PixelCount V9 algorithm, was categorized into three levels: weak (1), moderate (2), and strong (3). The final score for each area was determined by a weighted sum, in which the percentage of pixels in each category was multiplied by its corresponding intensity score. Based on the predefined protocol, the final scoring range was 100–300 [20]. Appropriate fields were selected from individual digital images and saved as TIFF files using the ImageScope image capture function.

### Monocyte isolation and differentiation

Human peripheral blood mononuclear cells (PBMCs) were isolated from buffy coats using SepMate tubes (Stemcell Technologies, Vancouver, BC, CA). The monocyte population was enriched by positive selection of CD14-labelled target cells using the human magnetic antibody cell sorting (MACS) system (Miltenyi Biotec, Bergisch-Gladbach, Germany), according to the manufacturer’s instructions. The CD14^+^ cells were initially cultured in RPMI1640 medium (Gibco, Waltham, MA, USA) supplemented with 10% heat-inactivated fetal bovine serum (FBS) (Omega Scientific, Inc, Tarzana, CA, USA), 100 U/mL penicillin, 100 U/mL (Gibco), and 2 mML-glutamine (Euroclone) for 24 hours at 37°C in 5% CO_2_. The supernatant was then harvested and the cells were incubated with fresh complete medium containing recombinant human M-CSF (20 ng/ml; R&D System, Minneapolis, MN, USA) for six days to differentiate them into non-polarized macrophages. M1 polarization was induced by supplementation with interferon-gamma (200 ng/mL; R&D System) and lipopolysaccharides from *E. Coli* (LPS, 1 ng/mL; Sigma-Aldrich, St. Louis, MO, USA) for 48 hours, whereas M2 polarization was obtained by supplementing cells with IL-4 (30 ng/mL) and IL-13 (30ng/ml), all from R&D System, for 48 hours. Macrophage polarization into M1 and M2 phenotypes was validated, as demonstrated in Supplementary Figure 1. To obtain medium conditioned by monocytes, M1, and M2 macrophages, cells were seeded at 3×10^5^in a 24-well plate (Corning Inc., Kennebunk, ME, USA) in RPMI-medium containing 0.5% FBS, 100 U/mL penicillin, and 100 U/mL streptomycin, and incubated for 24 hours at 37°C. After incubation, the supernatant was harvested and centrifuged, and cell-free supernatants were used for proliferation, invasion, scratch assays, and Proteome Profiler Analysis. The supernatants from monocytes, M1, and M2 cells were named monocyte-conditioned medium (CM), M1-CM, and M2-CM, respectively. All cells were negative for mycoplasma contamination (MycoAlert Mycoplasma Detection Kit, Lonza, Walkersville, MD, USA).

**Figure 1:**
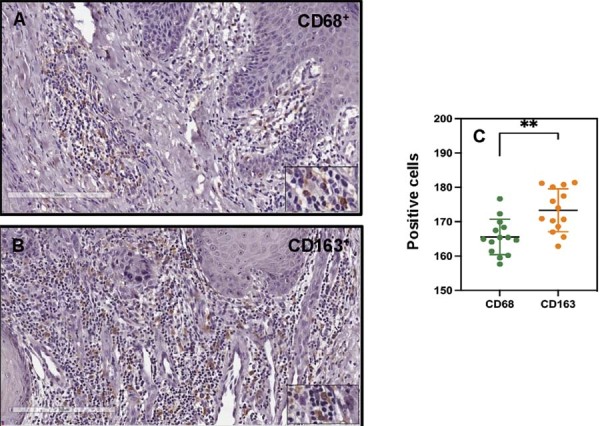
CD68 and CD163 expression in human squamous cell carcinoma. (A) Representative photomicrograph of a squamous cell carcinoma sample showing cells stained for CD68. (B) Representative photomicrograph of a squamous cell carcinoma sample showing cells stained for CD163. (C) Quantification of CD68 and CD163 expression in squamous cell carcinoma. The graph represents the mean ± standard error of CD68+ and CD163+ cells in squamous cell carcinoma samples. **P<0.01.

### Flow cytometry

Surface staining and flow cytometry were performed as previously described [16]. The following antibodies were used: anti-human CD68 Pacific Blue, anti-human CD64 PECY7, anti-human CD86 PE, anti-human CD206 Alexa-fluor 488, and anti-human HLADR APC. Corresponding mouse isotype controls were used for each antibody (BD Biosciences). Data were collected using a FACSCalibur (BD Immunocytometry Systems) and analyzed using CellQuest software (BD Biosciences).

### SCC cell lines

SCC-25 (CRL1628TM, HPV-negative) and Detroit 562 (HPV-negative) cells were purchased from ATCC (Manassas, VA, USA), cultured, and maintained according to the protocol described by Perri, et al.^21^ (2024). SCC-25 cells were cultured in Dulbecco’s Modified Eagle Medium (DMEM)/F12 (GibcoBRL, Waltham, MA, USA) supplemented with hydrocortisone (400 ng/mL), 10% heat-inactivated FBS (Omega Scientific, Inc, Tarzana, CA, USA), 50 IU/mL penicillin, and 50 μg/mL streptomycin (GibcoBRL). Detroit 562 cells were cultured in Eagle’s Minimum Essential Medium (EMEM) (GibcoBRL) with 10% FBS, 50 IU/mL penicillin, and 50 μg/mL streptomycin (GibcoBRL), and maintained at 37°C in 5% CO_2_. All cells were negative for mycoplasma contamination (MycoAlert Mycoplasma Detection Kit, Lonza, Walkersville, MD, USA).

### Proliferation assay

For each SCC cell line, 1×10^4^ cells/well were seeded in 96-well plates and allowed to adhere at 37°C in 5% CO_2_. After 24 hours, the cells were fasted in 0.1% bovine serum albumin (BSA) for 4 hours, and subsequently incubated with M1- or M2-CM. The proliferation was assessed by live-cell imaging (10× objective lens) using the IncuCyte ZOOM integrated software (Sartorius, Ann Arbor, MI, USA). At different time points, the software automatically calculated cell confluency. Values on the y-axis represent the fold change based on the baseline cell confluency without macrophage-CM at 0 hours. Methods were adapted from Perri, et al.^21^ (2024) with modifications as noted.

### Cell wound healing assay

SCC cell lines were seeded in an Essen Imagelock 96-well plate (1.5×10^4^ cells/well) and maintained until reaching confluence. After serum starvation with 0.1% BSA, a wound was made in the monolayer using a 96-well wound-maker tool with polytetrafluoroethylene (PTFE) pin tips (ESSEN BioScience), according to the manufacturer’s instructions. The scratched wells were then incubated with monocyte- or macrophage-CM at 37°C in 5% CO_2_. Live-cell images were taken every 2 hours for up to 48 hours with the IncuCyte ZOOM system, and wound closure was quantified with the integrated software. Experimental procedures followed the protocol established by Perri, et al.^21^ (2024).

### Cell invasion assay

SCC cell lines were fasted in 0.1% BSA and then plated (0.1×10^6^) on transwell inserts (8 μm) coated with Matrigel matrix, phenol red free (BD Biosciences, Franklin Lakes, NJ, USA), as previously described.^19^ The inserts were placed in 24-well plates containing 500 μL of cell-free macrophage-CM and incubated at 37°C for 48 hours. The cells attached to the bottom of the membrane were fixed with 4% paraformaldehyde, stained with 0.1% (v/v) crystal violet, washed, and imaged at 10× magnification using an inverted microscope (EVOS M5000, Invitrogen MA, USA). Cell counts were quantified using ImageJ (National Institutes of Health, Bethesda, MD, USA). Methods were adapted from Perri, et al.^21^ (2024), with modifications as noted.

### Reverse transcription-quantitative polymerase chain reaction (RT-qPCR)

Total RNA was extracted from SCC cell lines using Trizol (Life Technologies, Invitrogen, Carlsbad, CA, USA), and 1 μg of total RNA was reverse-transcribed into complementary DNA (cDNA), which was subsequently used for qPCR (Applied Biosystems Viia 7 instrument; Thermo Fisher Scientific). Target gene expression was normalized to housekeeping genes 18S or GAPDH. Relative gene expression was calculated using the standard 2^-^method. All primers for qRT-PCR were designed and synthesized by IDT Technologies (Coralville, IA, USA).

### Statistical analysis

Statistical significance was assessed using Student’s *t*-test for comparisons between two groups or one-way analysis of variance (ANOVA) for comparisons among three or more groups. Data are presented as mean ± standard error of the mean (SEM) or the mean ± standard deviation (SD). A P-value ≤.05 was considered statistically significant. The Prism 8.3 software program (GraphPad Software, San Diego, CA, USA) was used for statistical analysis.

## Results

### Macrophages in human squamous cell carcinoma lesions

Tumor-associated macrophages are a major component of myeloid cells in tumors. To distinguish macrophages from other inflammatory cells, they can be identified by the expression of CD68 and the human scavenger receptor CD163.^22^ In light of this, we investigated whether CD68 and CD163 expression could serve as prognostic markers in SCC. Analysis of CD68 protein levels in paraffin-embedded tissue sections revealed the presence of CD68^+^ cells in SCC samples (Figure 1A). CD163^+^ cells were detected in the peritumoral stroma, showed typical cytoplasmic staining, and were mononuclear infiltrated cells (Figure 1B). Immunohistochemical analysis further demonstrated higher immunoreactivity of CD163^+^ cells compared with CD68^+^ cells in SCC samples (Figure 1C).

### Effects of monocyte- and macrophage- conditioned medium on motility, proliferation, and invasiveness of SCC cell lines

**Figure 2:**
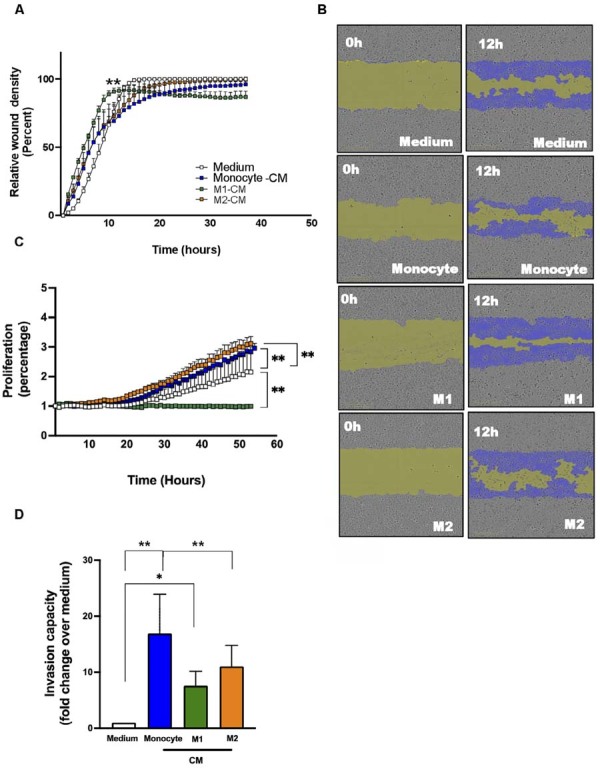
Motility, proliferation, and invasiveness of SCC-25 cells after exposure to monocyte- and macrophages-conditioned medium. (A) Relative wound density curve of SCC-25 cells over 40 hours as measured by IncuCyte analysis. (B) Representative images of scratch assays showing wounds immediately after scratching (0h) and after 12 hours in the presence of monocyte- and macrophage-CM (right panels) versus control medium (left panels). Scale bars represent 300µm. (C) Proliferation curve for SCC-25 cells in the presence of monocyte and macrophages-derived conditioned medium (M1-CM and M2-CM). Data are presented as mean ± standard error of the mean (SEM) from a single experiment and are representative of at least two experiments. (D) Transwell invasion assay for SCC-25 cells at 48 hours post-incubation with monocyte- and macrophage-CM. Data are shown as mean ± SEM of cells counted in five representative microscopic fields per membrane using the ImageJ software. *P <0.05; **P<0.01

The presence of different macrophage subsets in SCC patient samples led us to investigate the effects of conditioned medium obtained from human monocytes and monocyte-derived M1 and M2 macrophages on the behavior of two head and neck cancer cell lines. First, we assessed the cell motility and proliferation in the presence of CM-derived monocytes, M1, and M2 (Figure 2A) using real-time quantitative cell analysis. The confirmation of macrophage differentiation into M1 and M2 subsets (Supplementary Figure 1) provided a basis for interpreting downstream functional assays. SCC-25 cell motility increased over time for all stimuli; however, at 6–12 hours post-treatment, a significant increase in motility was observed only with M1-CM (Figure 2A and 2B), reaching a plateau at 24 hours. Additionally, no significant differences were found in the SCC-25 motility after M2-CM and monocyte-CM treatment (Figure 2A–B), which plateaued at 24 hours. M2-CM and monocyte-CM significantly enhanced SCC-25 cell proliferation (Figure 2C), whereas M1-CM-treated cells maintained a stable proliferation index. We further assessed whether monocyte- and macrophage-CM modulate SCC-25 invasiveness (Figure 2D). The invasive capacity of SCC-25 cells increased after treatment with all tested stimuli (Figure 2D). Specifically, SCC-25 cells stimulated with Monocyte-CM (12.4-fold±6.98-fold), M1-CM (7.6-fold±2.55-fold), and M2-CM (11.05-fold±3.74-fold) displayed elevated invasiveness (Figure 2D). These results showed that monocyte- and M2-CM enhanced proliferation and invasion, while M1-CM had no significant effects on the proliferation of SCC-25 cells. The findings also suggest that monocytes, M1, and M2 macrophages exert distinct biological effects on SCC-25 cells.

**Figure 3:**
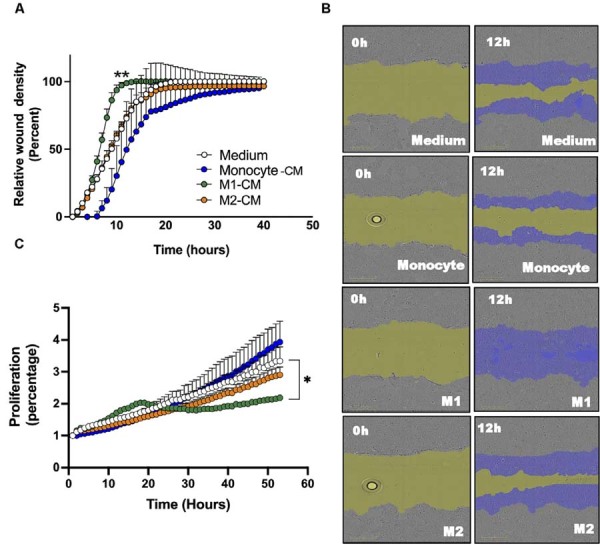
Influence of monocyte, M1, and M2 macrophage-conditioned medium on Detroit 562 cell motility and proliferation. (A) Relative wound density curves for Detroit 562 cells over 40 hours as measured by IncuCyte analysis. (B) Representative images of scratch assays showing wounds immediately after scratching (0 hours) and after 12 hours in the presence of monocyte- and macrophage-derived conditioned medium (right panels) versus control medium (left panels). Scale bars represent 300 µm. (C) Proliferation curve for Detroit 562 cells in the presence of monocyte- and macrophage-derived conditioned medium. *P <0.05. Data are shown as mean ± SEM from a single experiment and are representative of at least two experiments.

Having observed that monocyte- and macrophage-CM modulate SCC-25 cell functions, we investigated whether similar effects occur in Detroit 562 cells, which are derived from metastatic sites. Detroit 562 and SCC-25 cells showed comparable motility responses to monocyte- and macrophage-CM, and the motility of Detroit 562 cells increased over time for all stimuli. At 6–12 hours post-treatment, a significant increase in Detroit 562 cells motility was also observed with M1-CM, while monocyte-CM reduced cell motility (Figure 3A). Motility following M2-CM treatment was not significantly different from medium-stimulated controls (Figure 3A–B) and reached a “plateau” at 20 hours post-culture. In the proliferation assays, M1-CM significantly decreased Detroit 562 cell proliferation compared with controls (Figure 3C), whereas monocyte- and M2-CM-treated cells showed no differences in proliferation relative to the medium. Notably, Detroit 562 cells displayed minimal or absent invasiveness compared with SCC-25 cells (data not shown). In our study, Detroit 562 cells were unable to invade Matrigel or collagen matrices after 24–48 hours incubation with CM or 10% FBS (positive control) (data not shown).

**Figure 4:**
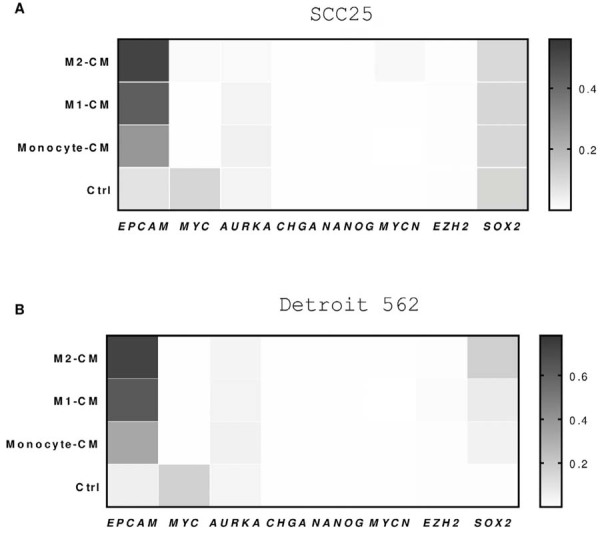
Impact of monocyte- and macrophage-derived conditioned medium on tumor plasticity gene expression in SCC cell lines. mRNA expression was determined by quantitative polymerase chain reaction (qPCR) (mean ± SEM, n=3) in SCC-25 (A) and Detroit 562 cells (B) after 24 hours of exposure to monocyte- and macrophage-derived conditioned medium. Heatmaps illustrate the relative expression of genes associated with epithelial-mesenchymal transition (*EPCAM, SOX2*), stemness (*NANOG, MYC, EZH2*), and neuroendrocrine differentiation (*CHGA, AURKA, MYC*).

To verify whether monocyte- and macrophage-CM modulate the tumor plasticity of SCC cells, we analyzed the expression of genes associated with epithelial-mesenchymal transition (*EPCAM, SOX2*), stemness (*NANOG, MYC, EZH2*), and neuroendocrine differentiation (*CHGA, AURKA, MYCN*). SCC-25 cells expressed markers of epithelial-mesenchymal transition, and both monocyte- and macrophage-CM upregulated *EPCAM* expression (Figure 4A). Among stemness-related genes, only *MYC* was expressed, and its expression was significantly downregulated by monocyte- and macrophage-CM (Figure 4A). SCC-25 cell lines did not express most neuroendocrine differentiation markers except for *AURKA*, whose expression was not significantly affected by monocyte- or macrophage-CM (Figure 4A). Similarly, we analyzed mRNA expression of selected genes linked to tumor plasticity and the neuroendocrine phenotype in Detroit 562 cells after 24 hours of incubation with CM. The expression of *CHGA, NANOG, MYCN,* and *EZH2* were not detected in Detroit 562 cells (Figure 4B). *EPCAM* and *SOX2* expression was upregulated in Detroit 562 cells stimulated with monocyte- and macrophage-CM compared with controls (Figure 4B). We also observed a significant downregulation of *MYC* mRNA after CM from monocytes, M1, and M2 (Figure 4B). Moreover, Detroit 562 cells expressed *AURKA*, a neuroendocrine differentiation marker, but its mRNA levels were unaffected by the stimuli (Figure 4B). Overall, monocyte- and macrophage-CM induced *EPCAM* and *SOX2* expression in Detroit 562 cells, with M2-CM producing a stronger effect, suggesting a significant role in the regulation of these genes.

## Discussion

Tumor-associated macrophages are the predominant immune cells in the SCC tumor microenvironment.^23^ They are now understood to compromise multiple distinct populations with overlapping M1-like and M2-like features.^24^ A striking example of the diverse possible outcomes in cancer arises from the many facets of macrophage polarization.^25^ Macrophages display a broad functional spectrum and can change their behavior depending on the microenvironment. These phenotypes may be influenced by factors such as microenvironmental location, tumor stage, and cancer type.^26-28^ We conducted several experiments to determine the effect of secreted products from monocytes and/or macrophages on SCC cell lines.

In this study, a predominance CD163^+^ cells was observed in SCC samples, particularly in the peritumoral stroma. The levels of the human scavenger receptor CD163 are significantly elevated in M2-like macrophages.^29^ The presence of M1 and M2 macrophages on peritumoral inflammation areas, but not within the tumor stroma, was positively correlated with higher grades of oral tongue SCC, suggesting that the spatial distribution of TAMs within tumors impacts patient prognosis.^30^ The presence of both M1 and M2 macrophages in the TME creates a dynamic immune environment, often resulting in immune tolerance or suppression that favors tumor survival and progression. *In vivo*, macrophages rarely exhibit exclusively M1 or M2 activation patterns; rather, they exist along a continuum characterized by the co-expression of markers linked to both classical and alternative polarization. This remarkable heterogeneity highlights the complex and dynamic characteristics of the tumor microenvironment, in which multifaceted signaling interactions influences macrophage function. M2-like macrophages significantly promote tumor growth by enhancing angiogenesis, inhibiting anti-tumor immunity, and contributing to extracellular matrix remodeling. Conversely, whereas M1-polarized macrophages are typically associated with pro-inflammatory and anti-tumor responses, recent studies indicate they may also induce pathogenic inflammation that paradoxically supports malignancy.^31-33^ In various cancers, M2-macrophage infiltration at tumor site correlates positively with tumor cell proliferation, metastasis, angiogenesis, and immune regulation.^31,34^ Moreover, we found that M2-CM promoted motility, proliferation, and invasiveness of SCC-25 cells. Similarly, M2-CM increased the migration rates and directionality of two oral SCC cell lines (SCC-25 and Cal27).^35^ M2 macrophages are frequently characterized by high levels of TGF-beta IL-10, low IL-12, and are involved in anti-inflammatory responses, angiogenesis, and tissue remodeling.^10^ Such activities may contribute to extracellular matrix (ECM) remodeling and epithelial-to-mesenchymal transition (EMT) in tumors.^36^ Our results showed that M2-CM promoted *SOX2* expression in Detroit 562 cells, a transcript factor related to several malignant processes. Elevated *SOX2* expression is often associated with poor prognosis, higher tumor grade, and reduced overall survival in SCC patients.^37^ This modulation of gene expression suggests that macrophages can influence SCC cell plasticity, potentially affecting their metastatic potential and response to therapy.^38^

We observed that conditioned medium from M1 macrophages exhibited distinct effects on Detroit 562 and SCC-25 cell lines. Exposure to M1-CM might have anticipated invasion behavior in both SCC cell lines. However, M1-CM did not affect SCC cell proliferation, despite the well-established role of TAMs in secreting growth factors and cytokines that facilitate cancer cell growth.^39^ This finding may be attributed to various factors, including the intrinsic heterogeneity of tumors from diverse tissue types and the cell-type specificity of macrophage-tumor interactions. Additionally, these SCC cell lines could produce sufficient endogenous growth factors to promote proliferation independently from M1-derived signals. Although evading cell cycle arrest can enhance proliferation, growth may still be limited by intrinsic factors such as senescence, metabolic stress, or activation of DNA damage responses.^40^ Notably, Detroit 562 cells exhibited lower invasiveness than SCC-25 cells, suggesting inherent differences in invasive capacity potentially influenced by their distinct origins and the surrounding microenvironment mediators.^41^ In this context, our results revealed that M1-CM treatment significantly decreased the proliferation of Detroit 562 cells, contrasting with the higher proliferation observed in monocyte-CM-treated cells. This dichotomy highlights the complex role of macrophages plasticity in tumor biology, in which M1 macrophages may exert anti-proliferative effects, potentially via the secretion of inhibitory cytokines.^42^ The role of M1 macrophages in cancer is notably complex, as evidence points to both tumor-promoting and tumor-suppressing effects. Some studies have demonstrated that conditioned media from M1 macrophages can enhance the migration and invasion of neoplastic cells, including oral squamous cell carcinoma, via mechanisms involving GDF15 and ErbB2 phosphorylation.^43^ Conversely, research on other cancer, such as esophageal squamous cell carcinoma, indicates that M1 macrophages inhibit invasion and migration, which is associated with improved patient prognosis.^44^ This apparent paradox highlights that M1 macrophage activity is context-dependent and varies with the specific tumor microenvironment. Similarly, the influences of M1 macrophages on tumor cell proliferation are variable. While some studies report that M1 macrophages inhibit proliferation in cancers such as colon cancer, indicating a potential anti-tumoral function,^45^ others highlight that M1-derived factors may support tumor cell survival in certain contexts.^43^ Collectively, these findings emphasize the dualistic and nuanced role of M1 macrophages in cancer progression. Their capacity to both promote invasion and exert anti-tumoral effects appears to be strongly influenced by the characteristics of the specific tumor microenvironment and cancer type. Understanding this complexity is crucial to effectively harness M1 macrophages in cancer therapies.

Additionally, the analysis of gene expression related to epithelial-mesenchymal transition and stemness revealed that macrophage-CM upregulated *EPCAM*, a marker associated with epithelial characteristics, while downregulating *MYC* (c-Myc), a key stemness gene.^46^ M2-CM induced the highest levels of *EPCAM* expression. The ablation of *SOX2* has been associated with tumor regression.^47^ Interestingly, M2-CM regulated the expression of these genes in Detroit 562 cells, whereas no significant changes were found in constitutive expression in SCC-25 cells, reflecting the heterogeneity of SCC tumors. Among proliferation regulators, M1-CM did not alter *SOX2* expression in SCC-25 cells but decreased *MYC* expression. The role of c-Myc in cell proliferation is linked to its ability to promote progression from G1 and into the S phase of the cell cycle.^48^ It has been reported that c-Myc depletion inhibits proliferation of human tumor cells at various cell cycle stages.^49^ The differences in cell cycle responses to c-Myc downregulation observed in our results further highlight the heterogeneity of c-Myc as a regulator of SCC cell proliferation. Our results suggest a possible role for *MYC* in modulating tumor cells; however, therapies targeting *MYC* must consider potential toxicity to normal tissues.^50-51^ Furthermore, in Detroit 562 cells, macrophage-CM treatment significantly upregulated EMT-related genes, suggesting that macrophages can induce a more plastic and invasive tumor phenotype. Conversely, *MYC* was downregulated, indicating a potential shift away from a stem-like state in response to macrophage signaling.^52^ Lastly, we noted that Detroit 562 cells expressed only one marker of neuroendocrine differentiation, *AURKA*, and that monocyte- and macrophage-CM treatment did not significantly modulate its expression. These findings imply that, while macrophages can influence many aspects of tumor cell behavior, their effects on neuroendocrine differentiation markers may be limited. Although our model offers valuable insights into certain signaling pathways, it does not fully capture the complexity of the TME, as it cannot replicate direct cell-to-cell interactions or the intricate structure of the TME.^53^ Therefore, gene expression changes in this system should be interpreted with caution, since they do not incorporate the complex signaling networks and physical components present *in vivo*.^54^

## Conclusions

Our findings demonstrate that conditioned media from monocytes and macrophages significantly impact the proliferation, invasiveness, and gene expression of tumor cells, underscoring the importance of the tumor microenvironment in cancer progression. The distinct effects of monocytes, M1, and M2 macrophages highlight the complexity of macrophage interactions within the tumor microenvironment and their potential as therapeutic targets^41-55^. Understanding these dynamics may guide efforts to modulate macrophage activity and potentially improve outcomes for individuals with SCC. However, further studies are required to elucidate the particular processes by which monocytes and macrophage-derived factors impact tumor behavior and to assess the therapeutic implications of targeting these interactions.
